# Outbreak of invasive meningococcal disease caused by a meningococcus serogroup B expressing a rare *porA* genosubtype (19-54, 15), Spain, March to April 2024

**DOI:** 10.2807/1560-7917.ES.2025.30.44.2500222

**Published:** 2025-11-06

**Authors:** Raquel Abad, Carmen Navarro, Cristina García-Amil, Marina Montes, Alfredo Castañeda-García, Juan A Cuadros, Alicia Galar, Fernando Martin, Ester Mena, Sara Pérez de Madrid, Carmen Román, Marta Soler, Julio A Vázquez

**Affiliations:** 1 National Reference Laboratory for Meningococci, National Centre for Microbiology, Instituto de Salud Carlos III, Madrid, Spain; 2 Hospital Universitario Príncipe de Asturias, Madrid, Spain; 3 Clinical Microbiology and Infectious Diseases Department, Hospital General Universitario Gregorio Marañón, Madrid, Spain; 4 Instituto de Investigación Sanitaria Gregorio Marañón, Madrid, Spain; 5 Servicio de Epidemiología, Subdirección General de Epidemiología, Dirección General de Salud Pública de Madrid, Madrid, Spain; 6 Hospital Universitario Severo Ochoa, Leganes, Madrid, Spain; 7 Hospital Universitario de Guadalajara, Castilla-La Mancha, Spain; 8 Servicio de Epidemiología, Dirección General de Salud Pública, Consejería de Sanidad de Castilla-La Mancha, Spain; 9 National Centre for Epidemiology, Instituto de Salud Carlos III, Madrid, Spain; 10 ECDC Fellowship Programme, Field Epidemiology path (EPIET), European Centre for Disease Prevention and Control (ECDC), Stockholm, Sweden

**Keywords:** Invasive meningococcal disease, outbreak, genosubtype

## Abstract

In Spain during March–April 2024, an outbreak of invasive meningococcal disease (IMD) occurred in four young adults, exhibiting high case fatality with two deaths. Cases 1 and 4 were confirmed by isolation of *Neisseria meningitidis* from blood samples, while Cases 2 and 3 were PCR-confirmed from cerebrospinal fluid (CSF). Serogroup B meningococcus with identical characterisation (B: 19–54, 15: F5–1: ST-34, cc32) was identified for all cases; the outbreak strain genosubtype PorA_VR1: 19–54 had not been previously described. Potential coverage of the outbreak strain by available MenB vaccines could not be predicted by molecular tools, so bactericidal response to the 4CMenB vaccine against the outbreak strain was measured by human serum bactericidal antibody assay (hSBA), defining the strain as covered by the vaccine. Two different social events were involved in transmission of the outbreak strain. According to the national meningococcal disease surveillance protocol, an active search for close contacts of the cases was conducted by public health authorities and timely chemoprophylaxis and/or vaccination with 4CMenB vaccine was recommended to over 200 contacts. The evolution of meningococcal strains with genosubtype 19–54 should be closely monitored, as it might confer a greater transmission capacity.

**Key public health message**

**What did you want to address in this study and why?**

*Neisseria meningitidis* bacteria often reside in the nasopharynx without causing disease but occasionally invade the body and cause invasive meningococcal disease (IMD). In Spain, most IMD cases are sporadic and only a few outbreaks have been identified in the past. We describe an outbreak among four patients with IMD by an uncommon strain of serogroup B *N. meningitidis* in March–April 2024 in Spain.

**What have we learnt from this study?**

We compared the *N. meningitidis* genotypes of all four patients with IMD to identify the outbreak strain which harboured a rare genotype *porA*, and confirmed that the outbreak strain was covered by the 4CMenB vaccine through serological tests. Two of the four patients with IMD died, and 200 close contacts were identified and offered antibiotics and/or a vaccine.

**What are the implications of your findings for public health?**

Genomic investigation showed the acquisition of this new genosubtype appears to be limited to a small group for now, but the evolution of strains with this rare genosubtype should be closely monitored.

## Background

Invasive meningococcal disease (IMD) is a major cause of bacterial meningitis and septicaemia, characterised by high morbidity and mortality. The causative pathogen *Neisseria meningitidis* is a Gram-negative bacterium that colonises the human nasopharynx without causing disease (asymptomatic carriage) but occasionally can also invade the body causing IMD [1]. Meningococcal colonisation can last several months, with person-to-person transmission occurring via respiratory droplets or secretions [1]. Invasive meningococcal disease remains rare in European Union/European Economic Area (EU/EEA) countries (incidence rate of 0.3 cases/100,000 inhabitants in 2022) with a high case fatality rate that varies across age groups, being the highest in patients aged 50–64 years (19%), 1–4 years (12%) and < 1 years olds (10%) [2].

In Spain, as in other European countries, the IMD incidence rates fell notably during the COVID-19 pandemic due to the non-pharmaceutical containment measures [3]. Since 2024, IMD incidence in Spain has approached levels observed in the years before the pandemic. In other European countries, a faster rebound in the number of cases has been seen, which was generally associated with an increase of IMD observed in the adolescent and young adult population (aged 17–24 years) [4,5]. 

There are 12 described meningococcal serogroups, with six (A, B, C, W, Y and X) causing the majority of cases globally [6]. Serogroup B remains the predominant serogroup in the EU/EEA countries in 2022, accounting for 62% of cases, followed by serogroups Y (16%), W (10%) and C (6%) [2]. In Spain, the IMD incidence rate in the 2023 was 0.55 of 100,000 inhabitants, with a case fatality of 13.2%, in line with EU/EEA countries [7], and a predominance of serogroup B (69% of all notified cases) followed by serogroup W (15.5%), serogroup Y (8.4%) and serogroup C (4.2%) [8].

In Spain, the majority of IMD cases are sporadic and only a few outbreaks have been historically identified and analysed [9,10]. Meningococcal vaccination is funded by the National Health System in Spain. Although the vaccination scheme may vary in different autonomous regions, the consensus recommended by the Ministry of Health, and included in the national immunisation programme, includes 4CMenB vaccination for children at 2, 4 and 12 months of age, the MenC conjugate vaccine at 4 and 12 months, and the MenACWY conjugate vaccine at 12 years old [11].

## Outbreak detection

An outbreak of four IMD cases (aged 17–30 years) by a serogroup B strain occurred in March–April 2024 in Spain. The outbreak was initially associated with the attendance of Case 1 at a party (Event 1) in a town (Place 1). After Event 1, cases appeared in three additional locations (Place 2: Cases 1 and 2; Place 3: Case 3; and Place 4: Case 4). A strain with a very uncommon *porA* genosubtype (VR1: 19–54, VR2: 15) was detected in all four cases. Here, we analyse the characteristics of the outbreak, including a molecular analysis of strains belonging to the same clonal complex (cc) to which the strain was associated (cc32).

## Methods

### Case definition

The four cases were notified according to the case definition in Spain [12] which coincides with the European case definition [13], initially to the authorities of the Autonomous Communities (regional) where the cases appeared and almost simultaneously to the National Centre for Epidemiology. A confirmed case of IMD is defined as any person meeting the laboratory criteria, which are at least one of the following four: (i) isolation of *N. meningitidis* from a normally sterile site, or from purpuric skin lesions; (ii) detection of *N. meningitidis* nucleic acid from a normally sterile site, or from purpuric skin lesions; (iii) detection of *N. meningitidis* antigen in CSF; and (iv) detection of Gram-negative stained diplococcus in CSF.

### Case investigation

Cases were admitted and treated at different local hospitals. The clinical diagnosis was meningococcal sepsis for Cases 1 and 4 and meningitis for Cases 2 and 3. Cases 1 and 4 were confirmed by isolation of *N. meningitidis* from blood samples, while Cases 2 and 3 were confirmed only by PCR from cerebrospinal fluid (CSF). 

For each case, demographic data, clinical presentation, vaccination status, travel history, outcome and complications about each IMD case were collected by health officials together with the clinicians and the relatives of the cases.

### Microbiological investigation

*Neisseria meningitidis* isolates from Cases 1 and 4 and CSF samples from Cases 2 and 3 were sent to the Spanish Reference Laboratory for characterisation and microbiological investigation. 

Both *N. meningitidis* isolates were grouped by slide agglutination with specific polyclonal antibodies [14]. For Cases 2 and 3, an in-house real-time PCR assay was used both for confirmation but also for genogroup determination [15,16].

Molecular characterisation (including PorA, FetA, MLST and FHbp genotyping) for all four cases was carried out [14].

The minimum inhibitory concentrations (MICs) of penicillin G, cefotaxime, ceftriaxone, rifampin and ciprofloxacin were determined in the two available isolates by ETEST (bioMerieux). ETEST was carried out according to the manufacturer's instructions, and the EUCAST breakpoint definitions [17] were used for interpretation of the obtained susceptibility results.

The prediction of 4CMenB vaccine coverage of the outbreak-associated strain was performed by applying the genetic Meningococcal Antigen Typing System (gMATS) algorithm [18] and Meningococcal Deduced Vaccine Antigen Reactivity (MenDeVAR) index [19]. Sera from individuals vaccinated with the 4CMenB vaccine were used against the outbreak-associated strain by a human serum bactericidal antibody (hSBA) assay, as previously described [20]. 

### Genomic investigation

DNA from cultured isolates was extracted by using the QIAamp DNA Mini Kit (Qiagen). Multiplexed libraries were created with Nextera XT DNA Library Preparation Kits (Illumina) and sequencing was performed on NextSeq Illumina platform (Illumina). The short-read sequences obtained were de novo assembled using SPAdes algorithm [14].

The assembled genomes were uploaded to the PubMLST Neisseria database (https://pubmlst.org/neisseria) [18] and annotated using a gene-by-gene approach through the Bacterial Isolate Genome Sequence Database (BIGSdb) platform as described previously [19].

The BIGSdb Genome Comparator tool was used to compare the two available outbreak isolates to each other and to a panel of 33 Spanish isolates belonging to the same cc. Additionally, genomes available in the PubMLST database (accessed 28 May 2024) from the meningococcal strains with the same genosubtype of the outbreak strain were also included in the comparative analysis. The Genome Comparator Tool employs a ‘gene by gene’ approach comparing arbitrary, sequentially assigned, pre-indexed allele identifiers at each locus. The 1,329 *N. meningitidis* core loci (present in ≥ 95% of the isolates in the *Neisseria* pubMLST) corresponding to *N. meningitidis* cgMLST v3 scheme (a modification of the scheme first published by Bratcher et al. 2014 [21], in which some loci have been removed due to either not being really core or having internal stop codons in a significant subset of isolates [22]) was used for genome comparison. Resulting distance matrices, automatically generates based on the number of variable alleles in the 1,329 core loci, were visualised as Neighbor-Net networks with SplitsTree4 V4 (http://www.splitstree.org).

### Outbreak management

According to the national meningococcal disease surveillance protocol [12], an active search for close contacts of the cases was carried out by public health authorities. Close contacts were defined as social contacts (attendees to the Event 1 and 2), family and coworkers close contacts, and health care workers contacts. The definition of close contacts was broadened to include other individuals who may have had some contact with the cases were included, such as neighbours, attendees of the same sports club etc., after the appearance and death of the fourth case. Contacts were recommended chemoprophylaxis (single-dose ciprofloxacin 500 mg) and vaccination with 4CMenB vaccine as soon as possible. The recommended dose schedule for the 4CMenB vaccine for adolescents and adults is two doses with ≥ 4 weeks between doses.

## Results

On day 1 in March 2024, 13 people with an average age of 30 years met in Place 1 to celebrate at Event 1. The usual place of residence of these 13 individuals was in different locations in two different Autonomous Communities in Spain. On day 3, one of the Event 1 attendees (Case 1) developed symptoms of fever, odynophagia and diarrhoea. They were hospitalised on day 5 and died on the same day with a diagnosis of meningococcal sepsis.

On day 7, a cohabiting close contact of Case 1 who did not attend Event 1 (Case 2) developed symptoms of fever and odynophagia and was admitted to the hospital the same day with a diagnosis of septic meningitis. After receiving treatment, they were discharged from the hospital on day 21. 

Eight of the Event 1 attendees in Place 1 met for a holiday trip from day 14–16 (Event 2) in a different town (Place 5). On day 21, an Event 2 participant (Case 3) travelling with some of the Event 1 attendees showed symptoms of headache, fever and vomiting. They were admitted to the hospital on the same day with a diagnosis of meningococcal meningitis and subsequently discharged on day 27.

Finally, on day 34, a person (Case 4) whose only apparent connection was residing in a town (Place 4) close to Place 5 showed symptoms of fever, abdominal pain and diarrhoea. Case 4 was admitted in the hospital on the same day with a diagnosis of meningococcal sepsis, and died on day 35.

**Figure 1:**
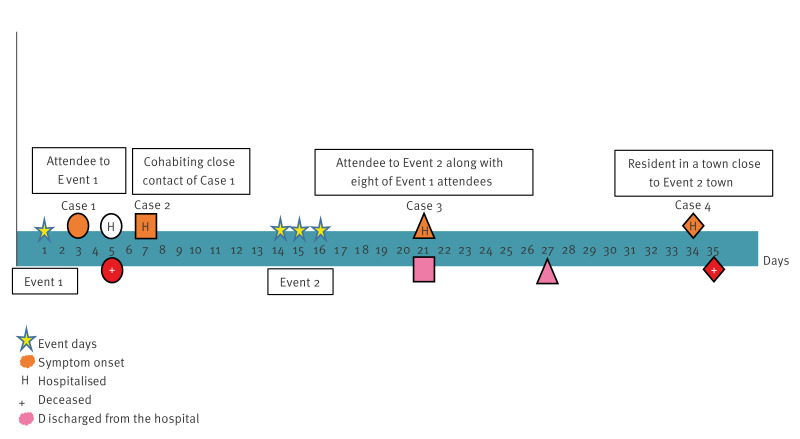
Timeline of events related to an outbreak of invasive meningococcal disease, Spain, March–April 2024 (n = 4 cases)

None of the cases were vaccinated against MenB. The timeline of IMD cases is shown in Figure 1.

Symptom onset of each case (indicated by different shape) is indicated in orange. Death (red +) or discharge (pink) is indicated.

### Microbiological analyses

For all four cases, a serogroup B meningococcus with an identical genotype was identified: PorA_VR1: 19–54, PorA_VR2: 15, FetA_VR: F5–1, ST-34 (cc32). The MICs of penicillin G, cefotaxime, ceftriaxone, rifampin and ciprofloxacin were determined in the two available isolates, and both were susceptible to all tested antibiotics. Molecular typing was performed on MenB vaccine antigens of isolates from Cases 1 and 4 (*fHbp*, *nhba* and *nadA*) and on CSF samples from Cases 2 and 3 (*fHbp*) as no isolates were available from these. Genes of all three antigens were present in the outbreak strain: *fHbp* allele 166, peptide 157, variant family 1, subfamily B; *nhba* allele 224, peptide 550; and *nadA* allele 1, peptide 1, variant 1.

None of the antigenic variants found in the outbreak strain was an exact match to the antigenic variants of protein-based MenB vaccines. The 4CMenB vaccine is composed by FHbp variant 1 (subfamily B) peptide 1, NHBA peptide 2, NadA variant 3 peptide 8 and PorA_VR2:4 [23], while rLP2086 vaccine includes two FHbp proteins: FHbp variant 1 (subfamily B) peptide 55 and FHbp variant 3 (subfamily A) peptide 45 [24].

The outbreak strain was defined as gMATS ‘unpredictable’ since although both PorA_VR2 (variant 15) and NadA (variant 1 peptide 1) were identified as predictors of non-coverage by gMATS, FHbp (peptide 157) and NHBA (peptide 550) were considered ‘unpredictable’. It should be noted that previously [18] it has been observed that, overall, 49% of gMATS ‘unpredictable’ strains were covered by MATS.

The reactivity of MenB vaccines against the outbreak strain also could not be predicted by the MenDeVAR Index since the strain contains antigens for which there were insufficient data or are yet to be tested in experimental studies.

Since potential coverage of the outbreak strain by any of the available MenB vaccines could not be predicted by molecular tools, hSBA was used to measure the ability of antibodies from serum of three vaccinated individuals (unrelated to the outbreak) to kill the outbreak strain, which is the accepted meningococcal vaccine surrogate of protection. A protective bactericidal response by the 4CMenB vaccine was observed, as the outbreak strain was killed in all the hSBA assays at a 1:128 dilution titre, above the 1:4 titre considered protective.

### Genomic investigation

The genomic relationship between the two outbreak isolates and 33 isolates belonging to the same cc32 available in the Spanish database, and the three genomes available in the PubMLST database from the meningococcal isolates with the same PorA_VR1: 19–54 was investigated. Supplementary Table S1 and S2 provide characteristics of the 33 cc32 isolates from the Spanish database and the three PorA_VR1: 19-54 isolates from the PubMLST database included in the genomic investigation. Core genome multilocus sequence typing (cgMLST) analysis was used for genome comparison and cluster detection.

**Figure 2:**
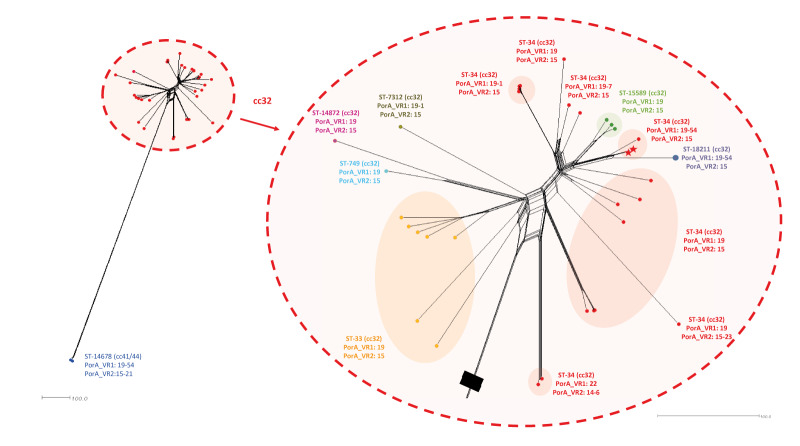
Neighbor-Net diagram comparing *Neisseria meningitidis* outbreak isolates, Spain, March–April 2024 (n = 2) and isolates from the Spanish database, 2017–2024 (n = 33) and PubMLST database, 2017–2023 (n = 3)

Genomic comparison of the 38 genomes confirmed that the two cases (Case 1 and Case 4) were nearly identical (only six allele differences among 1,329 loci). Both isolates cluster with the rest of the cc32 isolates and are very distant from the two carrier strains isolated in Australia with the same PorA_VR1: 19–54 but belonging to the cc41/44 (Figure 2). Among cc32 isolates, Case 1 and Case 4 isolates appear grouped with the other PubMLST database isolate PorA_VR1: 19–54, corresponding to a non-capsulated isolate from Spain in 2023 (57 and 51 allele differences from Case 1 and Case 4 isolates). The outbreak isolates also group with another PorA_VR1: 19–54 invasive isolate from a school outbreak in the Canary Islands in the same time period (unpublished data) but belong to the ST-18211 (86 and 80 allele differences from Case 1 and Case 4 isolates) (Figure 2).

The characteristics, including disease, capsular group and molecular typing (MLST, finetyping and MenB vaccine antigens) for the 2 outbreak and 33 cc32 *N. meningitidis* isolates from the Spanish database are provided in Supplementary Table S1; and for the three PorA_VR1: 19-54 *N. meningitidis* from PubMLST database in Supplementary Table S2.The scale bar represents a pairwise allelic difference of 100. The two *N. meningitidis* isolates from the Spanish outbreak are represented by red stars.

## Outbreak control measures

Cases 1 and 2 were cohabiting close contacts and developed symptoms 4 days apart. Thus, close contacts of these two cases in whom chemoprophylaxis and/or serogroup B vaccine should be recommended were traced together. For Cases 1 and 2, 65 close contacts were identified: 11 Event 1 attendees, 14 family contacts and 40 other individuals, who were either work contacts or healthcare personnel. All 65 received ciprofloxacin as chemoprophylaxis, and all contacts completed the treatment course; 57 (87.7%) received the 4CMenB vaccine.

Case 3 developed symptoms 18 days after Case 1 developed them. Twenty-one contacts were identified for Case 3: eight friends who attended Event 1 and 13 other close contacts. Twelve of these 21 received 4CMenB vaccine, taking into account that those eight attending the Event 1 already received vaccine after the second case appeared.

The appearance and death of the fourth case caused the level of alert to increase, so the definition of close contacts was broadened. A total of 122 Case 4 close contacts were identified, of which 120 received ciprofloxaci

## Discussion

We describe an outbreak of four IMD cases associated with serogroup B that occurred over a period of 1 month in 2024. Two social events appear to be involved in the transmission of the strain associated with the outbreak.

The reference laboratory received isolates from Cases 1 and 4, and a CSF sample from Cases 2 and 3. For all four cases, a serogroup B meningococcus with identical characterisation was identified: B: 19–54, 15: F5–1 ST34 (cc32). The genosubtype 19–54, 15 is very rare and PorA_VR1: 19–54 had not been previously described; the new variant was submitted to PubMLST for new allele assignment. Of strains showing the genosubtype 19–54, 15 appear grouped together in the phylogenetic tree, with only 6 allele differences in the 1,329 core loci analysed. There were 51 allele differences between the outbreak strain and one carrier strain isolated in Spain in 2023 characterised as ST-34 cc32 (same as the outbreak strain), and 80 allele differences with the strain associated with a school outbreak in Canary Islands characterised as ST-18211 cc32 (unpublished data).

The acquisition of this new VR1 variant 19–54 has occurred in a small group of isolates from cc32, and at least currently it appears to be limited to that small group. However, two strains were found in the PubMLST database (accessed: 28 May 2024) isolated from carriers in Australia in 2017 and 2018, which had already presented the variant VR1 19–54, but belonged to the clonal complex 41/44. This indicates that at least since that year, these strains acquired this new variable region 1 through an unknown mechanism and later strains of cc32 also acquired it, either from cc41/44 strains or by another route.

Clonal complex 32 is quite common in Europe, and constitutes a well-established hypervirulent lineage, although in Spain it represents only 10% of serogroup B strains since 2016 [14]. Apparently, the acquisition of VR1 19–54 could confer a greater transmission capacity since, in the same time period of the current outbreak, another school outbreak with two cases associated to another PorA_VR1: 19–54 meningococcal strain occurred in Spain (unpublished data). However, strains of both outbreaks, which belonged to the cc32, were from a different ST (ST-32 and ST-18211). The evolution of these strains with VR1 19–54 will have to be monitored to determine whether they occur as sporadic cases or continue to be associated with outbreaks.

The high case fatality found in the outbreak (2/4 cases) is also striking and is another relevant aspect to analyse in the surveillance carried out to monitoring the evolution of these strains.

The identification of Case 3, who attended the Event 2 together with some to the Event 1 attendees, is notable. Although the Event 1 attendees received ciprofloxacin after Case 1, it is likely that the new meeting during Event 2 facilitated the re-entry of this strain into the social circle linked to both events, but it could be that the strain is circulating in nearby circles as well as in the central core. In fact, the connection point of Case 4 with the central circle of friends has not yet been found, except for geographical proximity, which probably supports the circulation of this strain in peripheral circles.

Given the age of the cases (17–30 years), this outbreak would coincide with the rebound in the number of adolescent and young adult cases observed in other European countries after COVID-19 pandemic containment measures [4,5].

Finally, following the outbreak recommendations of the current IMD surveillance protocols [12], the 4CMenB vaccine was offered to close contacts. However, the potential coverage of the outbreak strain by any of the available MenB vaccines (4CMenB and rLP2086) could not be predicted by molecular tools. The bactericidal response of antibodies from serum of 4CMenB vaccinated individuals against the outbreak strain was measured by human serum bactericidal antibody assay (hSBA), defining the outbreak strain as covered by the vaccine. The evidence that microbiological laboratories can offer in predicting the coverage of available vaccines shows their importance for outbreaks control.

Because the Spanish Reference Laboratory only receives between 70% and 80% of IMD confirmed cases in the country, a limitation of the study would be that other previous IMD cases by a strain harbouring this new *porA* genosubtype may have occurred without being reported. In addition, a meningococcal carrier survey in cases and contacts might have provided information about the circulation of the outbreak strain.

## Conclusion

This outbreak highlights the importance of IMD microbiological surveillance in the detection and control of outbreaks, in addition to early clinical suspicion, antibiotic treatment and follow up of contacts. Molecular characterisation is a key aspect to the study and control of these types of outbreaks. In this outbreak, molecular characterisation for all four IMD cases allowed the identification of the outbreak strain, which resulted in the public health authorities recommending 4CMenB vaccination to control the outbreak.

## Data Availability

Genome assemblies of *Neisseria meningitidis* strains were deposited in PubMLST database (https://pubmlst.org/neisseria) under the PubMLST ids: 81847, 98338, 98339, 98362, 98363, 98368, 98375, 98409, 98414, 98487, 98506, 101924, 127209, 131885, 132969, 139961, 148183, 150081, 151013, 166810–166825.

## Supplementary Data

458000 Supplement
