# Supplementary material for: Outbreak of invasive meningococcal disease caused by a meningococcus serogroup B expressing a rare porA genosubtype (19-54, 15), Spain, March to April 2024
Source: Euro Surveill. 2025 Nov 6;30(44):2500222. doi: 10.2807/1560-7917.ES.2025.30.44.2500222 (PMC12595293; doi:10.2807/1560-7917.ES.2025.30.44.2500222)
Supplement: Supplement [file 25-00222_ABAD_Supplement.pdf]

This supplementary material is hosted by *Eurosurveillance* as supporting information alongside the article "*Outbreak of invasive meningococcal disease caused by a meningococcus serogroup B expressing a rare porA genosubtype (19-54, 15), Spain, March to April 2024*" on behalf of the authors who remain responsible for the accuracy and appropriateness of the content. The same standards for ethics, copyright, attributions and permissions as for the article apply. *Eurosurveillance* is not responsible for the maintenance of any links or email addresses provided therein.

Supplementary Table S1. cc32 isolates from the Spanish database included in the genomic investigation.

Isolate	Year	Disease	Capsular Group	MLST		Finetyping antigens			MenB vaccines antigens		
				ST	CC	PorA_VR1	PorA_VR2	FetA_VR	FHbp peptide	NHBA peptide	NadA peptide
22450	2017	invasive	B	34	32	19-1	15	F5-1	1	707	1
22451	2017	invasive	B	34	32	19-1	15	F5-1	1	707	1
22559	2017	invasive	B	34	32	19-1	15	F5-1	1	707	1
22560	2017	invasive	B	34	32	19-1	15	F5-1	1	707	1
22561	2017	invasive	B	34	32	19-1	15	F5-1	1	707	1
22595	2017	invasive	B	34	32	19	15	F5-1	1	3	1
22636	2017	invasive	B	34	32	19	15	F1-24	1	3	211
22648	2018	invasive	B	749	32	19	15	F5-1	61	120	1
22656	2018	invasive	B	33	32	19	15	F1-80	145	3	1
22899	2018	invasive	B	33	32	19	15	F5-1	1	3	-
22981	2018	invasive	B	34	32	19-7	15	F1-24	1	3	1
23207	2019	invasive	cnl	15589	32	19	15	F5-1	494	550	1
23260	2019	invasive	B	34	32	22	14-6	F4-28	1	109	1
23270	2019	invasive	B	34	32	22	14-6	F4-28	1	109	1
23312	2019	invasive	B	14872	32	19	15	F5-1	1	120	3
23371	2019	invasive	B	33	32	19	15	F5-1	1	3	1
23414	2019	invasive	B	33	32	19	15	F5-1	1	3	1
23453	2020	invasive	B	33	32	19	15	F5-1	16	3	1
23485	2020	invasive	cnl	34	32	19	15	F5-1	1	3	-

23552	2020	invasive	B	33	32	19	15	F5-1	1	3	1
23629	2020	invasive	B	7312	32	19-1	15	F1-96	1108	194	215
23725	2021	invasive	B	34	32	19	15	F5-1	1	3	1
23748	2021	invasive	B	34	32	19	15-23	F5-1	1	1757	1
23795	2022	STI	cnl	34	32	19	15	F5-1	1	20	1
23796	2022	carrier	cnl	34	32	19	15	F5-1	1	20	1
23867	2022	invasive	B	34	32	19-1	15	F5-1	1	707	1
23914	2022	invasive	cnl	15589	32	19	15-79	F5-1	1	550	1
23936	2023	invasive	B	33	32	19	15-81	F5-1	1	3	1
23949	2023	carrier	B	34	32	19	15	F1-3	-	550	1
23970	2023	carrier	cnl	34	32	19	15	F5-1	628	3	1
24069	2023	invasive	cnl	34	32	19	15	F5-1	1	20	1
24150	2023	invasive	B	15589	32	19	15	F5-1	1	1810	1
24272	2024	invasive	B	34	32	19-54	15	F5-1	157	550	1
24297	2024	invasive	B	34	32	19-54	15	F5-1	157	550	1
12218	2024	invasive	B	18211	32	19-54	15	F5-1	1072	550	1

Labelled in red the 2 outbreak isolates

- No peptide because frameshift, internal stop codon.

Supplementary Table S2. PorA VR1: 19-54 isolates from the PubMLST database (accessed May 28, 2024).

Isolate	Year	Disease	Capsular Group	MLST		Finetyping antigens			MenB vaccines antigens		
				ST	CC	PorA_VR1	PorA_VR2	FetA_VR	FHbp peptide	NHBA peptide	NadA peptide
MB35410	2017	carrier	NG	14678	41/44	19-54	15-21	F1-6	62	1696	-
MB65015	2018	carrier	NG	14678	41/44	19-54	15-21	F1-6	62	1696	-
H03-RESP-018	2023	invasive	cnl	34	32	19-54	15	F5-1	218	550	1

- No peptide because frameshift, internal stop codon.
